# Unsupervised clustering of SARS-CoV-2 using deep convolutional autoencoder

**DOI:** 10.1186/s44147-022-00125-0

**Published:** 2022-08-17

**Authors:** Fayroz F. Sherif, Khaled S. Ahmed

**Affiliations:** 1 Computers and Systems Department, Electronics Research Institute, Cairo, Egypt; 2 Biomedical Department, Faculty of Engineering, Benha University, Benha, Egypt

**Keywords:** SARS-CoV-2, Unsupervised clustering, Deep learning, Convolutional autoencoder, Spike protein, Lineages

## Abstract

SARS-CoV-2’s population structure might have a substantial impact on public health management and diagnostics if it can be identified. It is critical to rapidly monitor and characterize their lineages circulating globally for a more accurate diagnosis, improved care, and faster treatment. For a clearer picture of the SARS-CoV-2 population structure, clustering the sequencing data is essential. Here, deep clustering techniques were used to automatically group 29,017 different strains of SARS-CoV-2 into clusters. We aim to identify the main clusters of SARS-CoV-2 population structure based on convolutional autoencoder (CAE) trained with numerical feature vectors mapped from coronavirus Spike peptide sequences. Our clustering findings revealed that there are six large SARS-CoV-2 population clusters (C1, C2, C3, C4, C5, C6). These clusters contained 43 unique lineages in which the 29,017 publicly accessible strains were dispersed. In all the resulting six clusters, the genetic distances within the same cluster (intra-cluster distances) are less than the distances between inter-clusters (*P*-value 0.0019, Wilcoxon rank-sum test). This indicates substantial evidence of a connection between the cluster’s lineages. Furthermore, comparisons of the K-means and hierarchical clustering methods have been examined against the proposed deep learning clustering method. The intra-cluster genetic distances of the proposed method were smaller than those of K-means alone and hierarchical clustering methods. We used T-distributed stochastic-neighbor embedding (t-SNE) to show the outcomes of the deep learning clustering. The strains were isolated correctly between clusters in the t-SNE plot. Our results showed that the (C5) cluster exclusively includes Gamma lineage (P.1) only, suggesting that strains of P.1 in C5 are more diversified than those in the other clusters. Our study indicates that the genetic similarity between strains in the same cluster enables a better understanding of the major features of the unknown population lineages when compared to some of the more prevalent viral isolates. This information helps researchers figure out how the virus changed over time and spread to people all over the world.

## Introduction

The SARS-CoV-2 virus, which started in China, has continued to spread worldwide, infecting more than 235 million people and causing more than 4.8 million deaths (according to a WHO epidemiological update on October 5, 2021). The SARS-CoV-2 virus is a spherical virion with a 30 kb positive-stranded RNA viral genome that is translated into structural and nonstructural proteins. The Spike (S) protein is the most important surface protein of SARS-CoV-2 and is made up of a linear chain of 1273 amino acids. The spike (S) contains several functional parts called domains that perform diverse biochemical activities such as signal peptides from 1 to 13 and two subunits: S1 and S2. The S1 subunit starts from amino acid 14 to 685 and primarily includes the receptor-binding domain (RBD), which identifies receptors, followed by the S2 subunit from 686 to 1273, facilitating membrane fusion. Thus, one of the most worrying aspects of the SARS-CoV-2 spike protein (S) is how it moves or mutates over time as the virus evolves. Some of these mutations may change the biology of the spike and affect the virus’s transmissibility. Viruses change all the time, and SARS-CoV-2, the virus that causes COVID-19, is no exception. While SARS-CoV-2 mutations occur at a slower pace than other viruses such as influenza [1] and HIV [2], these genetic changes occur throughout time and can develop new variants with distinct features found in infected people as the pandemic progresses. The development of numerous prevalent SARS-CoV-2 variants in human populations may resist existing prevention and treatments [3]. Thus, genomic surveillance played a significant role in responding to the epidemic. As the COVID-19 vaccines become available and are used, genetic modifications of the SARS-CoV-2 monitoring processes are essential. Therefore, the FDA has been aware of the SARS-CoV-2 viral alterations and their possible impact on humans.

As a newly discovered virus, it is essential to consider SARS-CoV-2 genetic diversity, its evolutionary history, and possible transmission pathways from its natural reservoir to people. Most research has examined features of SARS-CoV-2 development and strain diversity in the real world using phylogenetic trees [4–6]. A phylogenetic tree is a graph that depicts the evolution of biological organisms based on their genetic similarities [7]. The distances between things represent the degree of their connections. However, when population genomic datasets expand in size, phylogenetic analysis using simply pairwise genetic distances is unable to reveal the full population’s structure. By clustering related entities into clusters and finding the number of key subtypes or clusters, it becomes simpler to comprehend the population’s primary features. Usually, entities have been clustered using the distance matrix and the bifurcations between the branches of the phylogenetic tree’s leaves. However, as the number of entities increases, it becomes more difficult to separate the clades in the phylogenetic tree directly and properly. Additionally, alignment-based methods such as BLAST and the Burrows-Wheeler Aligner (BWA) have been used to classify genome sequences. Such methods are based on labeling viral genes. Methods like BLAST have been very good at finding sequence similarities. However, when these methods are used to look at thousands of complete genomes, they take a lot of time to run [8]. They say that the alignments assume that the genes are homologous, which means that they all have the same continuous structure. It is not always the case.

Clustering approaches have been extensively employed as a useful supplement to phylogenetic study, including tree building [9], ancestral connection identification [10], evolutionary rate estimate [11], gene evolutionary processes research [12], and population structure analysis [13]. The clustering of SARS-CoV-2 lineages into subgroups is essential to biologists for many reasons [14]. Clustering representation offers a simplified understanding and analysis of the high-dimensional and large-scale biological data. Also, scientists can differentiate mutations that are only present in developing lineages with changes that affect viral biology. Moreover, the clustering process may significantly minimize sequence set redundancy and downstream analysis and storage expenses. Clustering procedures must be stable and resilient, and they must allow for information compression compared to non-clustered representations.

### Deep clustering

Clustering algorithms have emerged as more productive and robust approaches to grouping items. Several clustering approaches have emerged and applied to different data types such as text, images, and biological sequences. From the perspective of clustering, the data is divided into subgroups (clusters) so that patterns in the same cluster are more similar to patterns in other clusters. Clustering is frequently used in unlabeled data to discover natural groupings. Recently, deep learning achieved excellent performance in various supervised learning applications. This success motivates some unsupervised deep clustering approaches for labeling and clustering unlabeled data. Deep clustering is a term that refers to grouping using a deep neural network-related technique [15]. There are many efficient approaches for combining deep feature learning and classical clustering, such as convolutional autoencoders (CAE) [16]. Convolutional autoencoders are neural networks that combine the best parts of convolutional neural networks CNN and autoencoders AE into a single network called a CAE neural network. CNN can quickly pull out important features from input examples. On the other hand, unsupervised AE is capable of encoding input instances into low-dimensional representations. Moreover, AE can properly reconstruct the input with a minimal reconstruction error from these representations. In a nutshell, CAE begins by automatically extracting features from input core data and reducing their dimensionality using under-complete fully convolutional autoencoders. It is well established that reducing the data dimension and clustering in the feature space rather than the data space improves clustering performance [17]. In the second stage, the weights of the deep network are repeatedly fine-tuned by optimizing feature learning and clustering assignment concurrently so that the network learns features that improve clustering effectiveness [15].

### SARS-COV-2 lineages and nomenclature systems

There are currently three nomenclature systems to classify and monitor SARS-CoV-2 genetic lineages: the Global Initiative on Sharing All Influenza Data (GISAID) [18], Nextstrain, and Pango [19]. Each system employs a scientific approach to classifying and naming lineages. All three methods were developed before variants of interest (VOIs) and variants of concern (VOCs) were identified. Due to the existence of numerous naming systems, the same variant may have many names, usually concurrently and without regard to the VOI and VOC characteristics. Therefore, it is difficult for individuals who are not specialists to connect such variations to scientific papers. Similarly, the employment of different nomenclature systems confuses health consultants, the public, and the media and inhibits a good link between all stakeholders’ ability to communicate effectively to make timely choices on public health issues. The WHO encouraged organizations that have published phylogenetic-based categorization and nomenclature systems for SARS-CoV-2 variations, as well as specialists in virological and microbiological nomenclature, to contribute to the development of a better naming scheme for VOCs and VOIs. Participants have proposed utilizing Greek alphabet letters, such as alpha, beta, gamma, and Delta, which will make communication with nonscientific audiences simpler and more practical. Table 1 illustrates the list of currently designated variants of concern (VOC) and their definitions in the three nomenclature systems.

**Table 1:** Currently designated variants ofconcern (VOC)

WHO label	Variant type	First detection	GISAID	Nextstrain	Pango
Alpha	VOC	UK	GRY (formerly GR/501Y.V1)	20I/501Y.V1	B.1.1.7
Beta	VOC	South Africa	GH/501Y.V2	20H/501Y.V2	B.1.351
Gamma	VOC	Brazil	GR/501Y.V	20J/501Y.V3	P.1
Delta	VOC	India	G/452R.V3	21A/S:478K	B.1.617.2

### Related works

The current section reviews artificial intelligence-based solutions that may supplement existing conventional ways of fighting COVID-19 in global healthcare systems. Using artificial intelligence (AI) approaches, chest X-rays (CXR), and CT scans of COVID-19-suspected patients may assist in the diagnosis of COVID easily and quickly. A chest X-ray is one of the quickest techniques for identifying COVID-19 disease. Compared to other diagnostic techniques, X-ray pictures are regarded as a fast and cost-effective diagnostic technique. In recent years, in response to the need for speedy and precise analysis of CXR images, computer-aided diagnostic (CAD) tools have been created to help clinicians interpret a CXR picture [20–22] and introduced the present state and problems of computer-assisted diagnosis (CAD), machine learning (ML), and deep learning (DL)-based algorithms for CXRs as primary modalities for COVID detection. They analyzed several CXRs with COVID-19 and achieved 95.8% classification accuracy using the VGG16 architecture. Reference [23] suggested a network structure with DenseNet for feature extraction and a DL model called DenseCapsNet to identify COVID-19 from chest X-ray images with 98% accuracy. However, the difficulty with chest X-rays is that they cannot reliably discriminate soft tissues, and hence cannot be input into AI models for an all-around assessment. CT scans may be utilized to overcome this. The AI model learns by itself to discriminate COVID CT scans from non-COVID CT scans after reviewing a series of pictures. Several studies [24, 25] demonstrate significant success in the use of AI and deep learning (DL) algorithms for effective illness identification from chest CT images.

In parallel to this, the modeling approaches available to the deep learning community have grown significantly, some of which are already beginning to affect genomics. Deep neural networks (DNN) may increase prediction accuracy by identifying complicated and important features. Here, we outline deep learning modeling methods and their current uses in genomics. Recent publications have used deep learning for purposes such as viral prediction, viral host prediction, and prediction of a viral segment. For a more comprehensive examination of deep learning in genomics, we refer to a recent article in [26]. Here, Table 2 lists several state-of-the-art studies that used DNNs to analyze viral genome sequences. The objective of each study is described in the table along with the DNN employed, the input, the output, and the accuracy of the results. In addition to the biological disciplines and the input and output of the DNN, as shown in the third column of Table 2, the DNNs used in those references focus on using the advantages of convolutional neural networks (CNN) or long short-term memory (LSTM) in combination with a fully connected layer to improve classification and similarity score prediction accuracy.

**Table 2:** Summary of state-of-the-art references

Ref	Objective	DNN	Input	Output	Accuracy
[27]	Viral classification	CNN + FC	The whole genomic sequences of a virus	Different viral classes	96.7% (with noise) 98.7% (without noise)
[28]	Viral host prediction	RC-CNN and RC-LSTM	Contigs of a genome	Human or nonhuman host	91.7% CNN 86.3% LSTM
[29]	Viral classification	Stacked sparse autoencoder (SSAE)	Image representations of the complete genome sequences	Different classes	98.9% and 100%
[30]	Predicting the mutation rate of SARS-CoV-2	LSTM-RNN	Complete genome	Mutation rate calculations	(RMSE) of 0.06 in testing and 0.04 in training
[31]	Predicting the similarity score of the genome of “SARS-CoV-2” with other viruses	CNN + LSTM	Genome sequence	Similarity score with other viruses	99.27%

Based on DL approaches [27], offered a way to aid in the identification of SARS-CoV-2 during testing. A CNN architecture with four layers was used in order to extract the properties of the viral genomes and categorize SARS-CoV-2 as a member of the coronavirus family. The CNN received as input the whole genomic sequence of a virus. The nucleotides’ mapped numerical values were *C* = 0.25, *T* = 0.50, *G* = 0.75, and *A* = 1.0, respectively. Missing entries were assigned the value of 0.0. Experiments demonstrated that the CNN could accurately identify sequences even when noise was introduced to the genome, with accuracies ranging from 0.9674 (with noise) to 0.9875. (Without noise). The scientists also found a unique sequence for the SARS-CoV-2 virus based on their findings. This sequence was presented as a potential primer for PCR testing.

In [28], a method for classifying viruses utilizing contigs (fragments of the genome sequence) and two distinct reverse-complement (RC) neural network architectures (RC-CNN and RC-LSTM) was described. Additionally, these models were applied to the SARS-CoV-2 virus. Their model is trained to differentiate between viruses that infect humans and viruses that infect other chordates (nonhuman). The authors show that it is not easy to find the negative (nonhuman) class, which shows that the host-related signal is strong, and that the learned decision boundary is a very good way to tell human viruses from other DNA sequences.

Coutinho et.al. [29] proposed an alignment-free approach based on the stacked sparse autoencoder technique for classifying genomic sequences of the SARS-CoV-2 virus at different taxonomic levels (realm, family, genus, and subgenus). They investigated the use of a k-mers picture representation of the whole genome sequence, which facilitates the usage of genome sequences of any length and permits the use of fewer network inputs. The findings were presented using the confusion matrix for the validation and test sets, as well as the ROC curve for the validation set. All studies had accuracy rates ranging from 98.9 to 100%. These results demonstrate the relevance of the stacked sparse autoencoder approach for genome categorization issues.

Pathan et.al. [30] discussed the nucleotide mutation rate and pattern in the codon mutation set in this study. An RNN-based LSTM model has been developed to forecast the future rate of mutation in a COVID-19-infected individual’s body. In addition, they have described an LSTM-RNN model for time series prediction based on the nucleotide mutation rate of patients and forecasted the future mutation rate of the 400th patient. The root-mean-square error (RMSE) for this model is 0.06 in testing and 0.04 in training.

Rani et.al. [31] calculated the similarity score between the genome of “SARS-CoV-2” and the genomes of other viruses, including SARS-CoV, MERS-CoV, HIV, and HTLV. Working on the CNN- and LSTM-based “genome similarity predictor” model, which is used to classify genomes and predict the “SARS-CoV2” and other viruses’ “genomic similarity score.”

A recent study in [32] used deep embedding clustering [33] to group 16,873 strains. Six clusters on each continent have a distinct geographical distribution. This research analysis is restricted since more than 60% of SARS-CoV-2 strains are from the UK and USA. Africa and South America provide less than 2% of all strains. Sampling biases impact parameter estimation and clustering outcomes.

On the other hand, representing genomic data in unconventional ways has long been welcomed by researchers; for example, in recent work, the genome sequence was shown as a picture based on chaotic game representation to analyze diverse biological aspects [34]. On the other hand, a collection of 56 viral protein sequences from coronavirus, influenza, and Ebola were investigated and categorized using their auditory patterns [35].

To clarify the main population structure of the virus, grouping these strains into clusters is necessary, as these clusters displayed the major types of the virus. The genetic similarities of coronavirus strains within the same cluster enable a better understanding of the major features of the unknown virus lineages when compared to some of the more prevalent viral isolates. Also, it may provide insights towards identifying an effective medicine for the treatment of the COVID-19 strain from previously found one. However, the review of relevant research works reveals a lack of studies attempting to determine the population clusters of the “SARS-CoV-2” using deep learning approaches.

To the best of our knowledge, there were no deep clustering approaches have been used in combination with protein’s physical and chemical characteristics for COVID-19 population clustering based on unsupervised deep learning. Also, there was no mention of this method on other datasets in relevant papers. In this study, we aim to identify the main clusters of SARS-CoV-2 population structure based on convolutional autoencoder (CAE) trained with numerical feature vectors mapped from coronavirus Spike peptide sequences.

We begin by transferring the input sequences of coronavirus Spike protein into physiochemical features through the Proter software package [36]. The encoder learns the input features and reduces their dimensionality into compressed numerical feature vectors suited for clustering. It then uses a reduction in reconstruction loss to try to rebuild the original signal from the compressed such that useful information is not lost during the decoding process. The K-means clustering is used to learn the compressed feature vectors for determining the clustering labels for each spike protein. The proposed method’s effectiveness is validated by comparison with other state-of-the-art techniques such as K-means clustering without CAE and hierarchical clustering methods, using our available datasets. Furthermore, we used T-distributed stochastic neighbor embedding (t-SNE) to show the outcomes of the deep learning clustering. In short, the SARS-CoV-2 population structure analysis in this work helps researchers learn more about how the virus has changed and spread through human populations around the world.

## Methods

### SARS-CoV-2 sample collection

A sufficient number of gene datasets, including the entire genome sequence of SARS-CoV-2, are already accessible in the National Center for Biotechnology Information (NCBI) GenBank [37]. NCBI gave labels to virus sequences based on where they were found and when they were found. And each sequence had all the information about where the genes were written on it. We filtered the gene sequence, date of collection, and sample country among the various entities. COVID-19-affected genes are extracted from the human body. Additionally, there are a few incomplete genes in this collection. Therefore, we screened them and only kept those that had the whole genome. The focus of this study has been on spike protein changes due to their critical role in human infection. We collected 29,017 protein sequences from the NCBI viral database [37] on May 18, 2021. The downloaded strains are from all over the world, each with an average length of 23000 amino acids. At this time, we identified 42 distinct viral lineages in the collected sample. These lineages are mostly A, B, and B.1 according to Pango nomenclature, in addition to alpha, beta, delta, and gamma lineages according to WHO nomenclature. The resulting FASTA format sequences were required to be unique and complete Spike protein isolations. In addition, we chose amino acid sequences over nucleotide sequences for inclusion in our study because they give more reliable results.

### SARS-CoV-2 proposed architecture

**Fig. 1:**
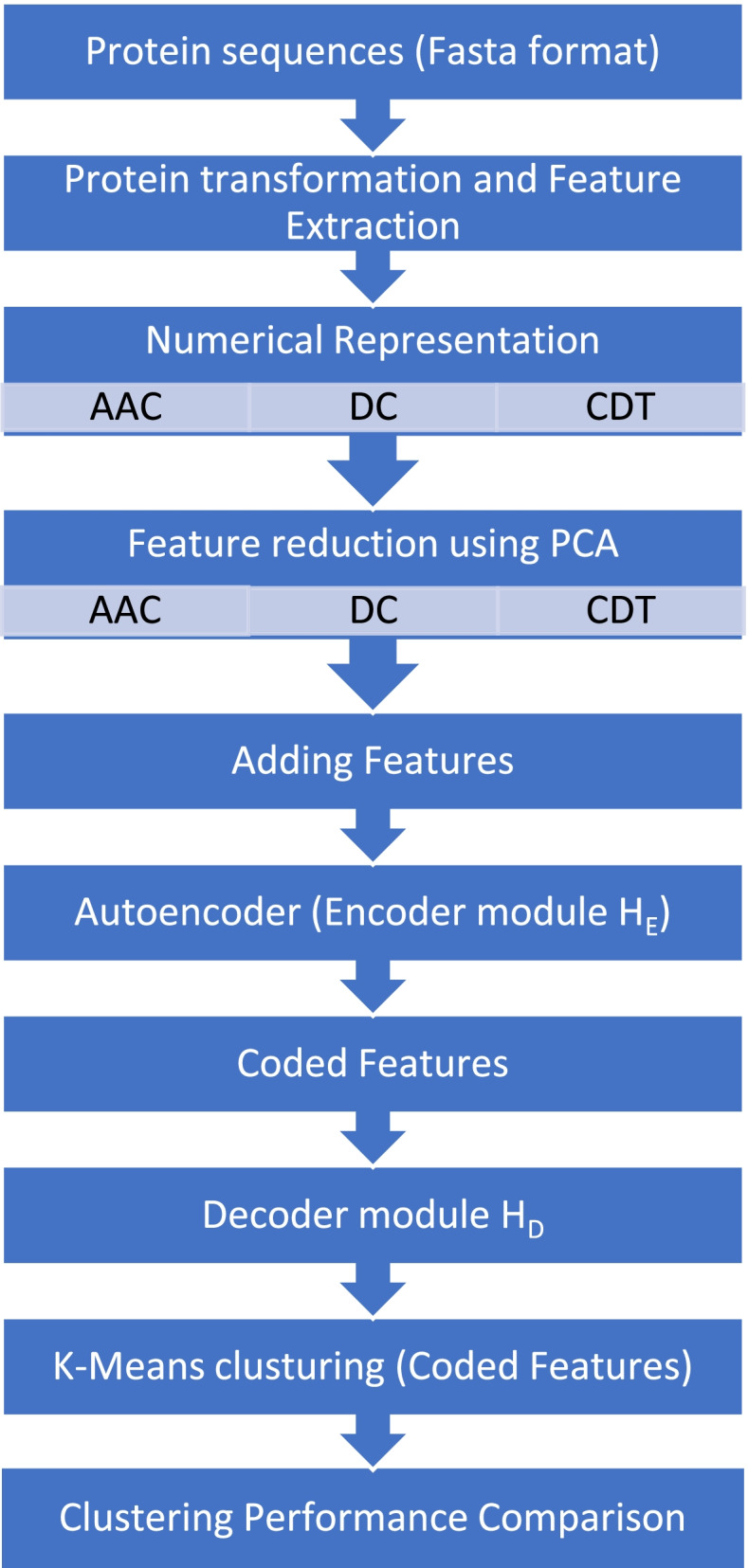
The proposed architecture

Figure 1 shows our proposed architecture that starts by transferring input sequences of Spike protein and reducing their dimensionality into relevant numerical feature vectors suited for clustering by the “Protr” software package in R. Then, we used principal component analysis (PCA) to produce a projection of a dataset before fitting a model. Using PCA can reduce the input numerical representation of each sample before applying CAE. The reduced input variables are fed to the convolutional autoencoders (CAE) network.

### Protein feature extraction

We aim to transfer the original data space (amino acid sequence) to a new space (numerical feature vectors) suited for clustering. The process of creating a useful numerical representation consists commonly of three major descriptors: amino acid composition (AAC), dipeptide composition (DC), and composition/transition/distribution (CTD) descriptors. The three descriptors are illustrated in detail in the next section. They are considered as numerical feature representations of proteins instead of their raw peptide data. By using this approach [38], the Spike protein sequences are converted into feature vectors, which include information on the existence, location, and order of k-tuples inside the protein sequence. With the Porter software package [21], protein sequences with comparable biological properties may be accurately classified, and links between spike sequences can be discovered. Here, we integrated amino acid decomposition (AAC), double decomposition (DC), composition-transition-distribution (CTD) features, and convolutional autoencoder-based clustering to discover the population clusters of the SARS-CoV-2 virus.

#### The Amino Acid Composition (AAC)

The amino acid composition (AAC) is one of the descriptors utilized in this work to convert the characters in a protein sequence to a numerical representation suitable for training deep learning algorithms. Basically, a protein sequence containing N amino-acid residues is often represented as a series, including the residue at the *i*^th^ position in the sequence. The labels i and j denote the location of amino acids in a sequence, whereas r and s denote the kind of amino acid (residue). AAC descriptor describes the makeup of each of the 20 naturally occurring amino acids found in protein sequences. The AAC is calculated as follows:

$$f(r)=\frac{N_r}{N}\kern1.5em r=1,2,3,\dots .,20.$$

where *N*_*r*_ denotes the number of r residues inside the sequence and *N* denotes the sequence’s entire length. The calculated descriptor is a named vector, each element of which is labeled with the name of the corresponding residue r.

#### Dipeptide Composition (DC)

The dipeptide composition (DC) descriptor is the rate of dipeptide amino acids within the protein sequences. DC gives 400 feature vectors, defined as follows:

$$f\left(r,s\right)=\frac{N_{rs}}{N-1}\kern1.5em r,s=1,2,3,\dots .,20$$

where *N*_rs_ denotes the number of dipeptide *rs* residues inside the sequence and *N* denotes the sequence’s entire length.

#### Composition/Transition/Distribution (CTD)

The CTD global protein sequence descriptors divide the amino acids into three groups according to their physical and chemical characteristics. The sequence is encoded by one of the symbols I, II, or III according to which group it belongs to. The class distribution pattern was defined for each peptide attribute (hydrophobicity, normalized van der Waals volume, polarity, polarizability, charge, secondary structure, and solvent accessibility) as shown in Table 3. This is how three descriptors were made for a certain attribute: composition (C), transition (T), and distribution (D). The composition descriptor set (C) contains information on the global percentage of each encoded group in the sequence. The resulting number of vectors is 21 (3 groups with 7 attributes each). At the same time, the transition descriptor set (T) reflects the percent frequency with which two classes transition along the sequence. There are 21 vectors in T (3 groups of 7 attributes each). The distribution set (D) is used to describe how each attribute is spread out in the sequence. Each characteristic has five “distribution” descriptions. They are percentages of the whole sequence for the first residue, 25%, 50%, and 75% of the whole sequence and 100% of the whole sequence for a certain encoded class [21]. Each vector is made up of three groups with seven attributes and five distribution places, which means a total of 105 feature vectors from the (D) descriptor only. The total number of scalar components in the parameter vector of these three descriptors is 21 (C) + 21 (T) + 105 (D) = 147 scalar components. In this case, 147 biochemical and physical features were used to show how each protein sequence is made up of different parts.

**Table 3:** The amino acid attributes and division of the amino acids into group

No.	Attribute	Group 1	Group 2	Group 3
1	Hydrophobicity	Polar {D/E/K/N/R/Q}	Neutral {A/G/H/S/P/T/Y}	Hydrophobic {C/I/L/F/M/V/W}
2	Polarizability	(0–1.08) {A/D/G/S/T}	(0.128–0.186) {C/E/I/L/N/P/Q/V}	(0.219–0.409) {F/H/K/M/R/W/Y}
3	Normalized van-der Waals volume	(0–2.78) {A/C/D/G/S/T/P}	(2.95–4.0) {E/I/L/N/Q/V}	(2.95–4.0) {F/H/K/M/R/W/Y}
4	Polarity	(4.9–6.2) {C/F/L/I/M/V/W/Y}	(8.0–9.2) {A/G/S/T/P}	(10.4–13.0) {E/D/H/K/N/Q/R}
5	Solvent accessibility	Buried {A/L/F/C/G/I/V/W}	Exposed {R/K/Q/E/N/D}	Intermediate {M/S/P/T/H/Y}
6	Secondary structure	Helix {E/A/L/M/Q/K/R/H}	Strand {V/I/Y/C/W/F/T}	Coil {G/N/P/S/D}
7	Charge	Positive {R/K}	Neutral {A/N/C/Q/G/H/I/L/M/F/ P/S/T/W/Y/}	Negative {E/D}

The numerical representations of Spike protein using the three major descriptors, AAC, DC, and CTD descriptors, are 20, 400, and 147, respectively. Thus, each Spike protein sample will be represented by 567 feature vectors obtained by concatenating the three descriptors. The numerical features we identified here will be learned using the convolutional autoencoder CAE to produce clustered groups of Spike proteins in terms of efficiency and effectiveness. These SARS-Cov-2 population clusters may cut down on sequence set redundancy, as well as the costs of further analysis and storage.

### Convolutional Autoencoder (CAE)

CAE can have multiple convolutions and pooling layers, each of which includes an encoder (which performs convolution and pooling operations) and a decoder (which performs un-pooling and deconvolution operations) [39]. The convolution layer in the encoder generates the *j*th feature map *h*^*j*^ from the input sample *x*_*i*_ as follows:

$${h}^j=\sigma\ \left({x}_i\ast {W}_{ij}^j+{b}^j\right)$$

where *x*_*i*_ is the input sample, ${W}_{ij}^j$ denotes the *j*th filter between input channel *i* and filter *j*, and *b*^*j*^ denotes the jth filter’s bias and *σ* is an activation function.

$${x}^j=\sigma\ \left({o}^j\ast {W}_{oj}^j+{c}^j\right)$$

where *o*^*j*^ is the *j*^th^ feature map and ${W}_{oj}^j$ is the *j*^th^ filter of the un-pooling layer o and j and *c*^*j*^ are the *j*^th^ output layer’s filter and bias, respectively.

An important part of input reconstruction is the loss function. This is what determines how good the reconstruction will be when it comes out. Sometimes, the loss in reconstruction is called the mean squared error (MSE), which is a way to measure how much difference there is between the original input and reconstructed input.

$${l}_{MSE}=\sum_{i=1}^M\sum_{j=1}^N{\left({x}_{ij}-{\hat{x}}_{ij}\right)}^2$$

where *x*_*ij*_ is the original input and ${\hat{x}}_{ij}$ represents the reconstructed input.

The bottleneck layer’s size has an impact on clustering performance. While one of the primary tasks of the proposed network design is automated feature extraction and dimensionality reduction, we discovered that having too few dimensions results in reduced clustering accuracy owing to increased reconstruction error. The network dimensions are determined by the problem’s complexity. For the most part, there is no set formula for determining the bottleneck dimension. Starting with a more comprehensive autoencoder and progressively decreasing the bottleneck dimension during pre-training until the reconstruction loss begins to noticeably increase is an empirical technique.

#### Implementation Algorithm

1. Select K as the number of SARS-COV-2 clusters (categories). Our objective was to have K viral clusters each represented by a centroid *μ*_k_, *k* = 1, 2, …. *K*.
2. Spike protein dataset *S* = {*s*_1_, *s*_2_, *s*_3_…*s*_*m*_} with *M* samples and *L* length were obtained and configured to be unique and complete into *K* clusters each represented by *μ*_k_, *k* = 1, 2, …. *K*.
3. Each Spike protein sample *S* is transformed into numerical representation using amino acid composition (AAC), dipeptide composition (DC), and composition/transition/distribution descriptors (CTD). Thus, the original input space *S* is transformed with descriptors mapping f: *S* → *X* into numerical vector representation with length *P*, where *P* < *L*.
4. For each sample, PCA is used to reduce the input numerical representation from *P to N*, where *N* < *P* before applying CAE.
5. A CAE model was designed to train the input matrix *X*_*M* × *N*_ where *M* is the total number of protein sequences in *S* and *N* is the length of the numerical feature vector. In which, *x*_ij_ is defined as the value of the *j*th descriptor of the *i*-th protein sample.
6. Creating and training K-means model using the coded features with different *k* number of clusters. The silhouette score explored that the top choice is six clusters.
7. Using trained K-means model for predicting clustering classes.

The suggested technique was implemented using Python and the Keras package [40], and all of the experiments were performed on the Google Colaboratory (Colab) [41], a free cloud-based Jupyter Notebook for training machine learning and deep learning models on CPUs, GPUs, and TPUs.

## Results and discussion

The focus of this study has been on spike protein changes due to their critical role in human infection. The dataset for this study included 29,017 samples of Spike protein, each with an average length of 23,000 amino acids. All the steps of transferring the input sequences of Spike protein into relevant numerical feature vectors suited for clustering are performed with the “Protr” software package in R. After data preprocessing, the input to CAE is a matrix with a total dimension of 567 × 29,017 (number of feature vectors × number of samples).

The input vector consists of 567 features, whereas the compressed representation has 45 features. This means that the output is twelve times smaller than the original input. Complex input characteristics impose typical unsupervised learning techniques, such as K-means and KNN, in this instance. However, including all characteristics would confound these algorithms. Applying an autoencoder, reducing input features, and extracting relevant data should come first. Then, an unsupervised learning method should be applied to the compressed form. Thus, clustering algorithms achieve excellent performance while producing more meaningful outcomes.

**Fig. 2:**
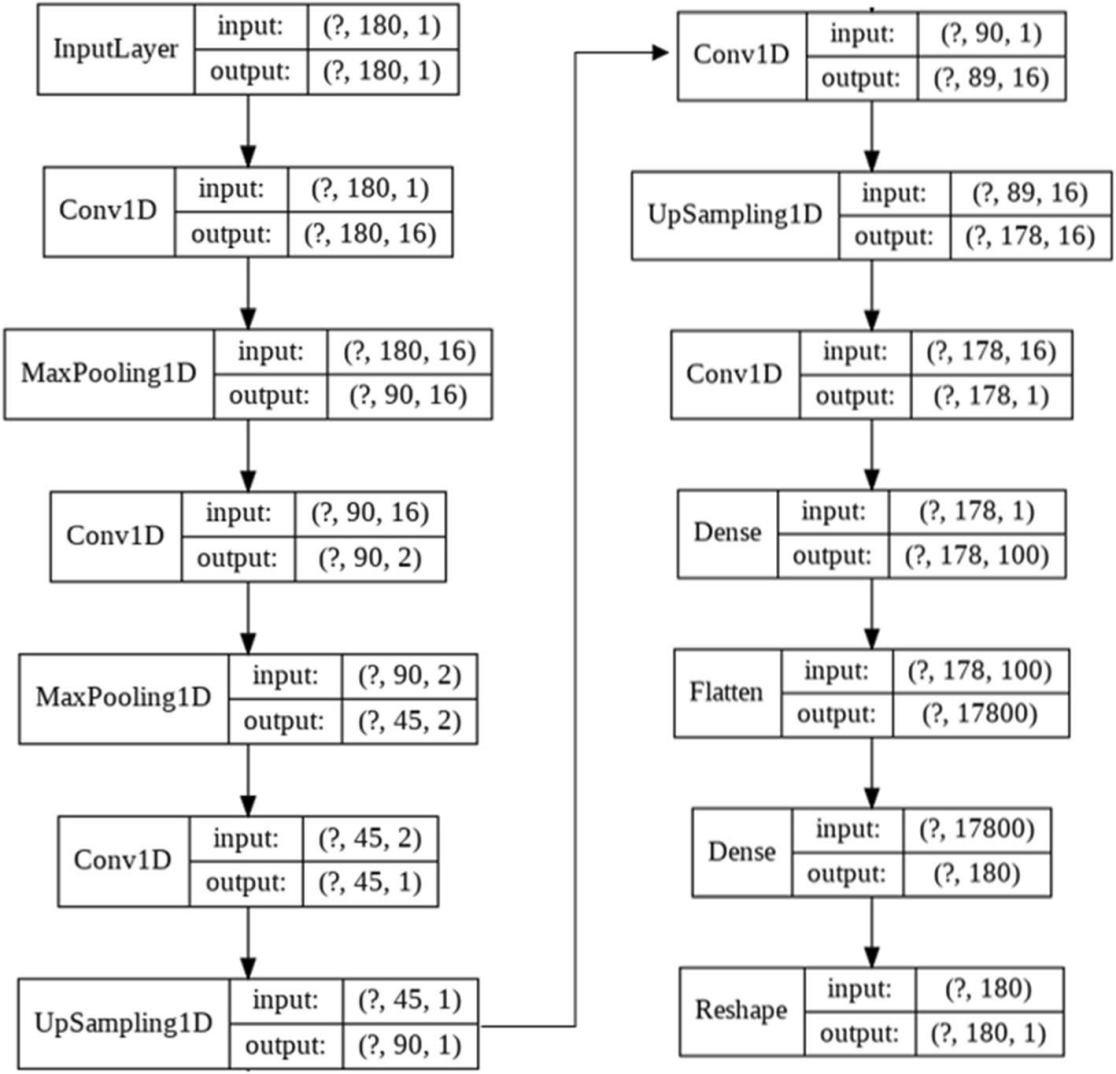
Model architecture using Keras visualization

Our CAE network is composed of two modules: an autoencoder hCAE = {encoder (hE), decoder (hD)} for learning protein features, and a K-means clustering model hCL for unsupervised clustering. The schematic representation of the CAE-based clustering is depicted in Fig. 2 where the CAE consists of 14 layers. The encoder component hE is composed of two convolutional layers and two max-pooling layers. Each convolutional layer generates a feature map through convolution between the input (or preceding layer’s feature map) and the learnable filter, followed by a max-pooling operation. The filter size in these convolutional layers is set to 3 × 3, and the number of filters is 16 and 2. The max-pooling method was introduced to determine the maximum value for each 2 × 2 area in each feature map. As a result, the size of the feature map in the current layer will be reduced to half that of the preceding layer only after the max-pooling procedure is completed. The frequently used ReLU (nonlinear activation function) is used to activate the convolved features. The last layer in the encoder is referred to as the bottleneck layer, whereas two deconvolutional layers comprise the decoder hD. The decoder module is trained to recover the original input from the encoder module using two upsampling and two deconvolution layers. The last two layers in the network are dense and flattened layers.

Tenfold cross-validation was employed. Additionally, 80% of the nine folds were utilized for training, while 20% were used for validation. We repeat the procedure of fine-tuning until the reconstruction error improves by no more than 6.4045e-04. The lower-dimensional feature vector produced from the encoder module hE has 45 features only. The autoencoder is trained for 100 iterations and then fine-tuned for approximately 6000 iterations. On a Google Colab [41], the entire pre-training and fine-tuning process took about 25 min, with the majority of that time spent on the fine-tuning step. After pre-training the autoencoder, we utilized the lower-dimensional feature space (coded vector) in K-means clustering. Thus, we do K-means clustering using 45 rather than 567 characteristics. This could help with the classification of unlabeled data.

### The effect of number of clusters

To pick an appropriate number of clusters and examine the impact of clustering, we used the number of clusters varied between two and eight to get diverse experimental findings. When working with higher dimensions, the silhouette score is beneficial for validating the clustering algorithm’s operation, since no visualization can be used to check to cluster when the dimensions exceed three. Additionally, we may utilize the silhouette score to determine the ideal cluster size. The silhouette coefficient sometimes referred to as the silhouette score is a statistic used to assess the quality of a clustering process. Its value is between −1 and 1. The complete experimental data are shown in Table 4 in terms of the number of clusters and the accompanying silhouette score. As shown in Table 4, when the number of clusters is around 6, the algorithm could generate the highest silhouette score which tends to be reduced slightly as the number of clusters increases. As seen in Table 4, the accuracy of clustering reduces steadily as the number of clusters increases. We may conclude that the best number of clusters is six in the preceding case since its silhouette score is higher than that of other clusters.

**Table 4:** The number of clusters and the corresponding silhouette score

No. of clusters	Silhouette score
2	0.519
3	0.727
4	0.752
5	0.825
6	0.885
7	0.874
8	0.878

The primary criterion for allocating a lineage to a cluster in this unsupervised study is a distance measurement, which includes the distance between items, the distance between the object and the cluster, and the distance between clusters. K-means groups things based on their distances from other objects and clusters.

**Fig. 3:**
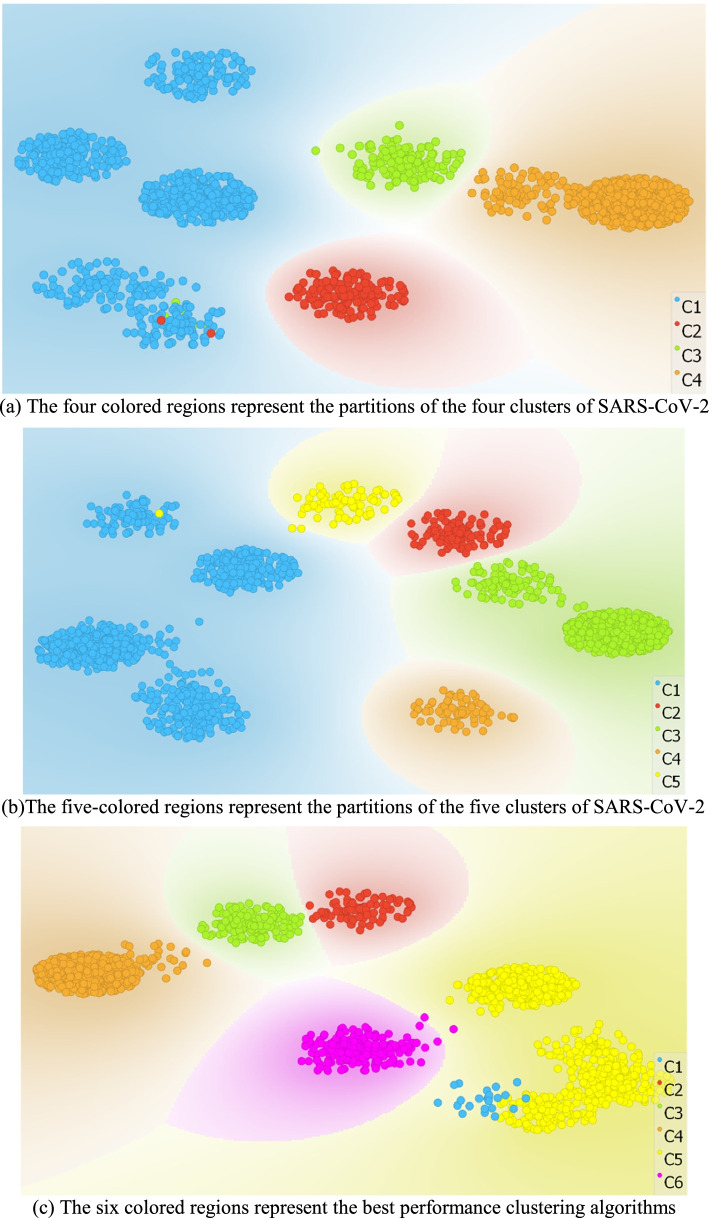
Comparing the t-SNE plots of the clustered SARS-CoV-2. **a** The four colored regions represent the partitions of the four clusters of SARS-CoV-2. **b** The five-colored regions represent the partitions of the five clusters of SARS-CoV-2. **c** The six colored regions represent the best performance clustering algorithms

Wilcoxon rank-sum test is a nonparametric statistical test used to compare two related samples, to see if their population mean ranks are different. The Wilcoxon test’s null hypothesis is commonly considered as equal medians instead of equal means. Rejecting the null hypothesis means that the median of two clusters differs, and they cannot be combined into a single cluster. Here we may infer that the median distance inside the cluster is lower than the median between clusters. In all six clusters, the genetic distances within the same cluster are less than the distances of inter-clusters (*P*-value 0.0019, Wilcoxon rank-sum test). This indicates substantial evidence of a connection between the cluster’s lineages. Following that, we used T-distributed stochastic neighbor embedding (t-SNE) to show the outcomes of the deep clustering. The strains were isolated correctly between clusters in the t-SNE plot. Comparative visualization of SARS-CoV-2 grouping into four, five, and six clusters was shown in Fig. 3. The visual results of clustered SARS-CoV-2 demonstrate that the best number of clusters was six as shown in the silhouette score.

Our clustering findings revealed six large SARS-CoV-2 population clusters (C1, C2, C3, C4, C5, C6) covered 43 unique lineages with 29,017 viral strains. In Table 5, we summarize the clustering groups across all lineages. The lineages are arranged within a cluster according to the highest distribution. The distribution of lineages in each cluster was determined by dividing the proportions of lineages from each cluster by the total number of strains within the cluster as shown in Table 5.

**Table 5:** The clustering groups across all lineages

Cluster	No. of lineages within the cluster	Lineage	Count of strains within the lineage	Distribution of lineage within the cluster
**C1**	1	A.1	1519	0.40
	2	A	458	0.12
	3	B	458	0.12
	4	B.1.617.2 (delta)	453	0.119
	5	A.3	450	0.118
	6	B.1.526	241	0.063
	7	A.2.2	62	0.016
	8	C.36.3	40	0.0105
	9	B.3	36	9.48 × 10^−3^
	10	A.2	29	9.48 × 10^−3^
	11	B.1.1.26	26	6.85 × 10^−^3
	12	B.28	24	6.3 × 10^−3^
		**Total**	**3796**	
**C2**	1	D.2	7544	0.995
	2	B.1	19	2.5 × 10^−3^
	3	A.1	14	1.84 × 10^−^3
		**Total**	**7579**	
**C3**	1	D.2	181	0.387
	2	C.37	135	0.288
	3	C.36.3	66	0.141
	4	B.1.1.207	35	0.074
	5	B.1.621	33	0.0705
	6	B.1.369	18	0.038
		**Total**	**468**	
**C4**	1	B.1	5841	0.433
	2	B.1.2	1619	0.12
	3	B.1.1	1065	0.079
	4	B.1.5	898	0.067
	5	B.1.243	644	0.047
	6	B.1.351 (beta)	566	0.041
	7	B.1.37	557	0.04
	8	B.1.595	516	0.037
	9	B.1.4	482	0.035
	10	B.1.320	427	0.031
	11	B.1.1.25	270	19.7 × 10^−3^
	12	B.1.1.7 (alpha)	155	11.3 × 10^−3^
	13	B.1.384	115	8.4 × 10^−3^
	14	D.3	115	8.4 × 10^−3^
	15	B.1.39	109	7.9 × 10^−3^
	16	B.1.1.26	107	7.8 × 10^−3^
	17	B.1.31	88	6.4 × 10^−3^
	18	B.1.268	84	6.1 × 10^−3^
	19	B.1.503	81	5.9 × 10^−3^
	20	C.35	48	3.5 × 10^−3^
	21	B.1.36	25	1.8 × 10^−4^
		**Total**	**13647**	
**C5**	1	P.1 (gamma)	974	1
		**Total**	**974**	
**C6**	1	B.1	372	0.145
	2	D.2	486	0.1902
	3	P.2	980	0.0407
	4	A.23.1	437	0.0145
	5	A.1	28	0.011
	6	B.1.525	219	7.436 × 10^−3^
	7	C.37	19	7.436 × 10^−3^
	8	B.1.3	14	4.7 × 10^−3^
	**Total**		**2555**	
**C1 + C2 + C3 + C4 + C5 + C6 =**				**29017**

The clustering results showed that the viral strains of B.1.617.2 (or delta), B.1.526, and ten additional lineages were grouped into the same cluster (C1). Our study indicates that the genetic similarity between these strains enables a better understanding of the major features of the unknown population lineages when compared to some of the more prevalent viral isolates. On the other hand, the C4 cluster is the largest in size, which comprises 13,646 strains of 21 different lineages. Moreover, the C4 cluster has a large set of well-known lineages such as B.1, B.1.1, B.1.1.7, B.1.320, B.1.509, and B.1.351 (according to Pango SARS-CoV-2 lineage nomenclature).

**Fig. 4:**
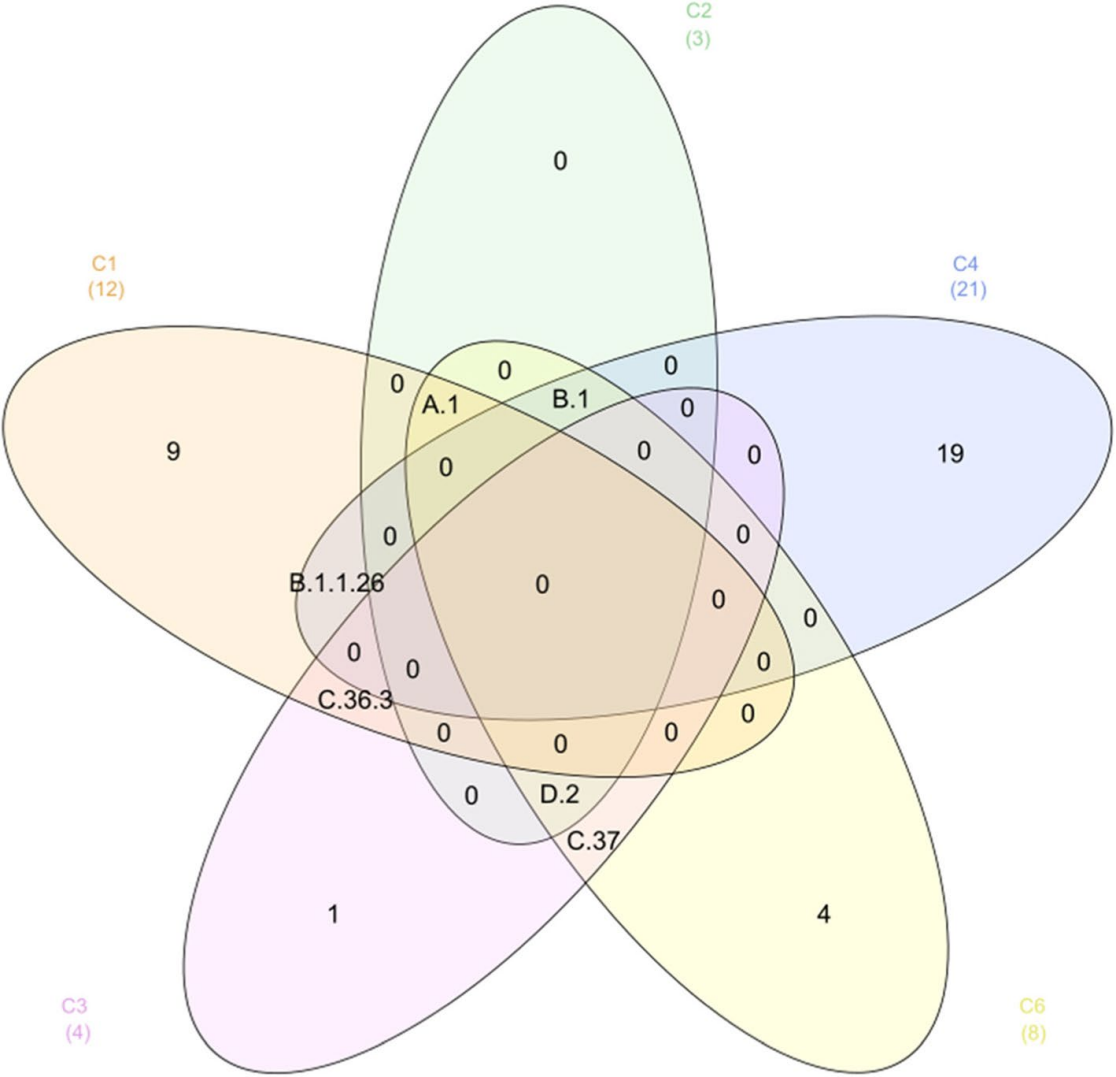
Venn diagram showing the total number of lineages expressed in the six clusters

Among these, the new variants B.1 (often referred to as the European variant), B.1.1.7 (usually referred to as the UK variant or Alpha), and B.1.351 (commonly referred to as the South African variant) have been generally classified as variations of concern (VOCs) due to indications of enhanced transmissibility, illness severity, and/or possibly reduced vaccination effectiveness. The second-largest cluster is C2, with a total number of strains reaching 7578. The most confirmed and concentrated lineage in this cluster was D.2, which occupied about 99.5% of the strains in the (C2) cluster. Also, D.2 is partially found in C3 and C6 clusters with a population frequency of 0.022 and 0.059, respectively (Table 5). Our results showed that the (C5) cluster exclusively includes gamma lineage (P.1) only, suggesting that strains of P.1 in C5 are more diversified than those in the other clusters. The majority of lineages (36 out of 43) were uniquely distributed in one cluster only, while the remaining lineages (6 out of 43) were significantly enriched in more than one cluster, as illustrated in Table 6. The Venn diagram depicts the total number of lineages exclusively expressed in the six clusters (Fig. 4).

**Table 6:** Lineages that develop in more than one cluster (overlapped cluster)

No.	Lineage	Total no. of strains	Overlapped clusters	The proportion of lineage in each cluster
1	D.2	8215	[C2]	0.918
			[C3]	0.022
			[C6]	0.059
2	B.1	6233	[C2]	3.04 × 10^−3^
			[C4]	0.937
			[C6]	0.059
3	A.1	1552	[C1]	0.979
			[C2]	0.02 × 10^−3^
			[C6]	0.018
4	B.1.1.26	133	[C1]	0.195
			[C4]	0.805
5	C.37	154	[C3]	0.876
			[C6]	0.123
6	C.36.3	106	[C1]	0.377
			[C3]	0.622

## Discussion

The deep learning models proposed in [27–29] were successful in detecting the viral genome in the host cell. The convolutional neural network described in [23] could accurately identify sequences even when noise was introduced to the genome, with accuracies ranging from 0.9674 (with noise) to 0.9875 (without noise). For the categorization of “SARS-CoV-2” from the provided genomic contigs into human and nonhuman classes [28], the model obtained 91.7% and 86.3% accuracy using CNN and LSTM, respectively. However, it does not identify similarities between the various genomic sequences of viruses. Therefore, it provides little insight into drug discovery. However, the accuracy of the deep learning model presented in [29] was greater than that of the model proposed in [27, 28] using the stacked sparse autoencoder approach, and the image representation of the whole genome sequence [31] calculated the similarity score between the genome of “SARS-CoV-2” and the genomes of other viruses, including SARS-CoV, MERS-CoV, HIV, and HTLV. Working on the CNN- and LSTM-based “genome similarity predictor” model, which is used to classify genomes and predict the “SARS-CoV2” and other viruses' “genomic similarity score.”

The comparison with the previous works reveals a lack of studies attempting to determine the population clusters of the “SARS-CoV-2” using deep learning approaches. To the best of our knowledge, there was only one study that used deep embedding clustering [32] to group 16,873 strains. Six clusters on each continent have a distinct geographical distribution. Their research analysis is restricted since more than 60% of SARS-CoV-2 strains are from the UK and USA. Africa and South America provide less than 2% of all strains. Sampling biases impact parameter estimation and clustering outcomes.

Our study’s contribution may be described as follows: This research proposes an efficient convolutional autoencoder model in combination with protein’s physical and chemical characteristics for COVID-19 population clustering based on unsupervised deep learning. The proposed model first implements the genetic feature transformation into physicochemical feature representation for preprocessing. Second, build CAE for learning protein features and unsupervised K-means for clustering. The proposed method’s effectiveness is validated by comparison with other state-of-the-art techniques such as K-means clustering without CAE and hierarchical clustering methods, using our available datasets. With the Wilcoxon rank-sum test, the average sum of the intra-cluster distances in the proposed deep clustering method was considerably smaller than that of K-means alone and hierarchical clustering methods (with a *P*-value < 0.05). Our findings agree with the literature [5, 32] in revealing that the best number of clusters that characterizes the SARS-CoV-2 population was six clusters.

Unfortunately, our study does not include the BA.1 Omicron variant of the SARS-CoV-2 lineage since it was first found in specimens collected on November 11, 2021, after we received our data. In a future study, we suggest using soft clustering methods to look at more viral genes, such as the BA.1 Omicron variant of SARS-CoV-2. The fundamental drawback of deep clustering is that it is an unsupervised task, so we cannot test its performance on real data. Hyper-parameter testing must rely on benchmark datasets, which raises serious doubts about whether deep clustering algorithms can be used in real-world contexts. Furthermore, our deep clustering technique does not explain why SARS CoV-2 protein sequences are grouped in a specific way, as opposed to alignment-based methods. As a result of this treatment, deep clustering models were viewed as “black boxes” with no explanation for their classification results. For our deep clustering models, we need far more training data than the alignment-based techniques, which only need one reference genome sequence for each class to work. A large number of instances must be provided to train a deep clustering model. Instead of using genetic data, a deep clustering method was used to put the SARS Cov-2 protein samples into groups with similar characteristics.

In future research, we may evaluate the effect of increasing the number of convolutional layers on model performance and training time. This will depend on the availability of computer resources, to discover a trade-off between model performance and training time.

## Conclusions

Understanding SARS-population CoV-2’s structure helps predict future infection risks. We analyzed 29,017 viral sequences to estimate population structure. We propose a convolution autoencoder-based deep clustering technique for grouping SARS-CoV-2 Spike proteins based on their physicochemical features instead of their genetic data. Our clustering found six significant SARS-CoV-2 clusters with 29,017 strains. We used other methods to verify deep learning clustering results. We first evaluated genetic distances within and between groups. In each of the six clusters, average intra-cluster distances are fewer than inter-cluster distances (*P*-value 0.0019, using the Wilcoxon rank-sum test). We utilized t-SNE to display the deep learning clustering results. The proposed method beats K-means and hierarchical clustering. Our analysis shows that the genetic similarity between cluster strains offers a better knowledge of the unknown population lineages compared to more ubiquitous virus isolates. The recommended technique can monitor and characterize circulating SARS-CoV-2 lineages. This helps professionals deliver better care, diagnose more accurately, and cure quicker.

## Data Availability

The datasets generated during and/or analyzed during the current study are available in the NCBI repository, NCBI Virus. Available at https://www.ncbi.nlm.nih.gov/labs/virus/vssi/#/sars-cov-2.

## Abbreviations

AAC: Amino acid composition
AE: Autoencoders
BWA: Burrows-Wheeler Aligner
CNN: Convolution neural network
CAE: Convolution autoencoder
CTD: Composition/transition/distribution
DNN: Deep neural networks
DC: Dipeptide composition
FDA: Food and Drug Administration
GISAID: Global Initiative on Sharing All Influenza Data
MSE: Mean squared error
NCBI: National Center for Biotechnology Information
PCA: Principal component analysis
RBD: Receptor-binding domain
S: Spike protein
t-SNE: T-distributed stochastic-neighbor embedding
VOCs: Variations of concern
VOIs: Variants of interest
WRST: Wilcoxon rank-sum test
WHO: World Health Organization
